# Composition and Antidiarrheal Activity of *Bidens odorata* Cav.

**DOI:** 10.1155/2013/170290

**Published:** 2013-10-27

**Authors:** Daniel Zavala-Mendoza, Francisco J. Alarcon-Aguilar, Salud Pérez-Gutierrez, M. Carmen Escobar-Villanueva, Miguel A. Zavala-Sánchez

**Affiliations:** ^1^Experimental Biology P.h.D Program, D.C.B.S. Universidad Autónoma Metropolitana-Iztapalapa, 09340 México, DF, Mexico; ^2^Departamento Ciencias de la Salud, D.C.B.S. Universidad Autónoma Metropolitana-Iztapalapa, Avenida San Rafael Atlixco No. 186, Colonia Vicentina, Iztapalapa, 09340 México, DF, Mexico; ^3^Departamento de Sistemas Biológicos, Universidad Autónoma Metropolitana-Xochimilco, Calzada del Hueso 1100, Colonia Villa Quietud, Coyoacán, 09340 México, DF, Mexico

## Abstract

The antidiarrheal effects of chloroform, methanol, and aqueous extracts of *Bidens odorata* Cav. were investigated at doses of 200 mg/kg on castor-oil-induced diarrhea. The chloroform extract of *B. odorata* (CBO) reduced diarrhea by 72.72%. The effect of CBO was evaluated on mice with diarrhea induced by castor oil, MgSO_4_, arachidonic acid, or prostaglandin E_2_. CBO inhibited the contraction induced by carbachol chloride on ileum (100 *µ*g/mL) and intestinal transit (200 mg/kg) in Wistar rats. The active fraction of CBO (F4) at doses of 100 mg/kg inhibited the diarrhea induced by castor oil (90.1%) or arachidonic acid (72.9%) but did not inhibit the diarrhea induced by PGE_2_. The active fraction of F4 (FR5) only was tested on diarrhea induced with castor oil and inhibited this diarrhea by 92.1%. The compositions of F4 and FR5 were determined by GC-MS, and oleic, palmitic, linoleic, and stearic acids were found. F4 and a mixture of the four fatty acids inhibited diarrhea at doses of 100 mg/kg (90.1% and 70.6%, resp.). The results of this study show that *B. odorata* has antidiarrheal effects, as is claimed by folk medicine, and could possibly be used for the production of a phytomedicine.

## 1. Introduction

Diarrheal diseases are among the most common gastrointestinal disorders and a major cause of morbidity and mortality in children under 5 years of age, particularly in underdeveloped countries. The reported global mortality due to diarrhea in children under 5 years is approximately 1.87 million, representing 19% of all childhood deaths [1].

Medicinal plants are commonly used in Mexico to treat diarrhea and other diseases; these plants are therefore considered potential sources of antidiarrheal drugs. Among them, *Bidens odorata* Cav., commonly known as “Mozoquelite” or “Aceitilla,” is used to treat gastrointestinal discomforts such as diarrhea [2, 3], as well as headaches and pain of the lumbar region [2, 4]. It has also been found that aqueous extracts of this plant have a diuretic effect [5]. However, there have been no reports regarding the antidiarrheal activity of this plant. For this reason, we decided to study the antidiarrheal activity of *B. odorata* and determine the composition of the active extract.

## 2. Experimental

### 2.1. Plant Material

*Bidens odorata* was collected at Fortín de las Flores, Veracruz State, in September of 2010. The material was authenticated by taxonomist José García-Pérez, and a voucher specimen (SLPM 21668) was deposited in the Herbarium Isidro Palacios of the Instituto de Investigaciones de Zonas Deserticas of the Universidad Autonoma de San Luis Potosi. The aerial parts of the plant (leaves and branches) were dried in the shade and powdered.

### 2.2. Extracts Preparation

In a two-litre flask fitted with a reflux condenser, 125 g of dried, powdered *B. odorata* was extracted at boiling temperature for 4 h with 1500 mL of solvent (chloroform, methanol, or water), after which the mixture was cooled to room temperature and filtered. The chloroform and methanol extracts were dried under vacuum in a rotatory evaporator followed by a vacuum oven at room temperature for 12 h. The aqueous extract was lyophilised. The yields of the chloroform, methanol, and aqueous extracts were 5.88, 10.40, and 9.04%, respectively.

### 2.3. Separation of CBO and Component Identification of Active Fractions

The CBO was separated by column chromatography using a column packed with silica gel (Kieselgel 60, 70–230 mesh ASTM) and prepared in hexane as the mobile phase. The polarity was increased with ethyl acetate, and 100 mL fractions were collected and compared by thin layer chromatography; subsequently, those with the same pattern were pooled, resulting in 6 fractions. The active fraction (F4) was a waxy semisolid with a melting point (mp) of 36°C that was obtained with a hexane/ethyl acetate ratio of 8 : 2. F4 was separated by column chromatography using a column packed with silica gel (Kieselgel 60, 70–230 mesh ASTM) and prepared in chloroform as the mobile phase. The polarity was increased with ethyl acetate, and 100 mL fractions were collected and compared by thin layer chromatography; subsequently, those with the same pattern were pooled, resulting in 7 fractions. The active fraction (FR5) had an mp of 34°C and was obtained with chloroform/ethyl acetate (9 : 1 v/v). 

### 2.4. Identification of the Active Compounds of F4 and FR5

The analysis of F4 and FR5 was performed on a gas chromatograph coupled to a mass spectrometer (Agilent Technology models 6890N and 5973) using a capillary column (DB-5HT) 15 m in length with a 0.25 mm internal diameter and 0.10 *µ*m film thickness. We used a temperature program starting at 100°C for 3 min, followed by a heating rate of 10°C per min to 320°C, at which point the temperature was maintained for 5 min. Splitless injection was used with a ratio of 1 : 100, and the injector temperature was 320°C. The spectra were determined at 70 eV, and the mass range was analysed from 33 to 800 *m/z*. The compounds were identified from mass spectra and compared to spectra reported in the NIST database (Wiley09/NIST11). 

### 2.5. Animals

Male CD1 mice (20–25 g) and male Wistar rats (180–250 g) from the Unidad de Produccion y Experimentacion de Animales de Laboratorio (UPEAL) of the Universidad Autónoma Metropolitana animal facility were housed in isolated cages under standardised conditions (dark/light 12/12 h) at 30°C and 50–55% humidity. The animals were supplied with rodent food (Pet Foods 5001) and water *ad libitum*. Prior to the study, the animals were submitted to a fasting period of 18–24 h with free access to water. 

All experiments were performed according to the current guidelines for the care of laboratory animals and the ethical guidelines for investigation in conscious animals [6].

### 2.6. Evaluation of the Effects of the Extract on Normal Defecation

Groups of five mice were individually placed in acrylic cages containing filter paper at the bottom [7]. CBO at a dose of 200 mg/kg or loperamide at 2.5 mg/kg was administered to the test groups, and a separate control group received only the vehicle. The total amount of faeces in each group was assessed every hour for the next 4 h. The percent reduction in the amount of faeces in the treated groups was calculated in comparison to the control animals (0% reduction).

### 2.7. Evaluation of Antidiarrheal Activity on Castor-Oil-Induced Diarrhea

Groups of 15 mice were administered the methanol or aqueous extract (200 mg/kg); loperamide (2.5 mg/kg); CBO (300, 200, 100, and 50 mg/kg); F4, a mixture of palmitic, linoleic, oleic, and stearic acid or each individual acid (100, 50, 25, 12.5, 6.25, 3.12, and 1.55 mg/kg); or vehicle (0.1 mL) 30 min before the administration of castor oil (4 mL/kg). All administrations were p.o. 

Following treatment, five animals of each group were placed separately in acrylic cages lined with filter paper that was changed every hour. The severity of the diarrhea was assessed each hour for 4 h. The total number of watery faeces excreted was scored and compared with the score from the control group. The total score of the diarrheic faeces of the control group was considered to be 100%, in accordance with the model described by Melo et al. [8], Litchfield and Wilcoxon [9], and Douglas et al. [10]. The results are expressed as a percentage of inhibition.

### 2.8. Evaluation of Antidiarrheal Activity on MgSO_4_-Induced Diarrhea

Groups of 15 mice, separated into boxes of five animals each, were administered the CBO (200, 100, and 50 mg/kg), loperamide (2.5 mg/kg), or vehicle (0.1 mL) 30 min before the administration of MgSO_4_ (4 mL/kg). The results are expressed as a percentage of inhibition.

### 2.9. Evaluation of Antidiarrheal Activity on AA- and PGE_2_-Induced Diarrhea

The antidiarrheal activity of CBO (100 and 200 mg/kg) and F4 (100 mg/kg) was evaluated on diarrhea induced with AA (3 mg/kg) and PGE_2_ (1 mg/kg). Three groups of 15 mice, separated into cages of five animals each, were administered the following treatments p.o.: group 1, CBO (200 mg/kg) or F4 (100 mg/kg); group 2, loperamide (2.5 mg/kg); and group 3, vehicle (0.1 mL). The treatments were administered 30 min after the three groups received i.p. AA or PGE_2_.

Following treatment, the groups were separated into subgroups of five animals, which were placed separately in acrylic cages lined with filter paper that was changed every half an hour. The severity of the diarrhea was assessed every 30 min for 2 h. The total number of watery faeces excreted was scored and compared with the score from the control group. The total score of the diarrheic faeces of the control group was considered to be 100%, in accordance with the model described by Melo et al. [8].

### 2.10. Small Intestinal Transit

The inhibitory activity of CBO on intestinal transit was tested using the following procedure [11]. Each group was administered vehicle, CBO (200 mg/kg), or loperamide (2.5 mg/kg) in a volume of 1.5 mL/animal, followed 30 min later by treatment with castor oil (4 mL/kg). A 2% suspension of graphite in 1.5% agar was orally administered to groups of 15 rats (1.5 mL/animal). At 30, 60, and 90 min after the administration of the graphite-agar suspension plus the castor oil, the rats were killed in groups of five, and the gastrointestinal tract was removed and opened. The distance travelled by the marker was measured and expressed as a percentage of the total length of the intestine from the pylorus to caecum. The results are expressed as percentages of intestinal transit and were subsequently compared to the results of the control group.

### 2.11. Evaluation of Effects on Ileum Contraction

Wistar male rats (300 g) were sacrificed by cervical dislocation. The abdomen was opened, and segments of ileum (10 cm proximal to the caecum) were flushed twice with aerated physiological salt solution (PSS) to remove the contents. The ileum was cut into segments approximately 1 cm long and placed in a 2 mL organ bath containing PSS with the following composition (mM): NaCl (118), NaHCO_3_ (25), KCl (4.7), KH_2_PO_4_ (1.2), MgSO_4_ (1.2), CaCl_2_ (2.5), and D-glucose (11). The organ bath was maintained at 36°C with aeration through bubbling with a mixture of 95% O_2_ and 5% CO_2_ (pH 7.4). Contractions of the ileum tissues were isometrically recorded through a force displacement transducer (Grass FT03) connected to a TBRS2 Grass polygraph. The tissues were allowed to equilibrate for 60 min, during which the PSS was changed every 20 min. The tissues were maintained under an optimal tension of 1 g prior to initiation of the experimental protocol. To expose the tissues to a single submaximal concentration of KCl (23 mM) and to elicit contractile activity, the ileum rings were bathed in a depolarisation solution (23 mM KCl) prepared by the equimolecular substitution of NaCl for KCl. Control contractile responses were considered to be two successive similar responses after a group of tissues (*n* = 6, two tissues per rat) were incubated with CBO dissolved in 20 *µ*L ethanol-dimethyl sulphoxide (1 : 1) or distilled water for 10 min. According to the model described by Estrada-Soto et al. [12], each tissue was stimulated with a different concentration of carbachol chloride (0.01, 0.1, 1.0, 10, or 100 *µ*M), and the amplitude was measured. 

The contractile response for the group treated with solvent alone was considered to be a response of 100% and was compared with the contractile response in tissues pretreated with 100 *µ*g/mL CBO, based on the method described by Estrada-Soto et al. [12].

### 2.12. Acute Toxicity

CBO was orally administered to groups of mice (*n* = 3) at single doses of 2500, 3750, or 5000 mg/kg. This range of doses used in mice followed the method presented by Lorke, D. and OECD/OCDE 420 [13, 14]. After administration, the animals were observed under open-field conditions for a 72-hour period. The number of animal deaths and signs of clinical toxicity were recorded. The median lethal dose (LD_50_) was calculated by the method described by Litchfield and Wilcoxon [9].

## 3. Statistics

The results are expressed as the means ± s.e.m. 

The differences between the mean values of intestinal transit were evaluated by Student's *t*-test, which is used in the analysis of equal variance of two groups with normal distribution [15]. The antidiarrheal activity and the effect on inhibition of ileum contractile activity were analysed using ANOVA and Tukey's post hoc test. These tests were used to compare the mean values of the antidiarrheal activity with the control group. Statistical significance was set at *P* < 0.05.

## 4. Results and Discussion

The pharmacological activities of the chloroform, methanol, and aqueous extracts of *B. odorata* at doses of 200 mg/kg on mice with diarrhea induced by castor oil are shown in Table 1. The results indicated that the aqueous and methanol extracts of *B. odorata* inhibited the diarrhea induced by castor oil by less than 50%. A previous study [10] reported the antidiarrheal activity of chloroform, methanol, and aqueous extracts at doses of 100 mg/kg and the % of inhibition was 45.6, 26.1, and 22.4%, respectively; for this reason we decided to test a higher dose (200 mg/kg).

However, CBO extract significantly diminished the induced diarrhea by 72.72% in this model. The results of CBO on castor-oil- or MgSO_4_-induced diarrhea are shown in Table 2. At a dose of 100 mg/kg, the extract reduced the diarrhea induced by MgSO_4_ by 84.85%; this antidiarrheal effect was decreased at a dose of 200 mg/kg_. _Additionally, it was observed that the greatest effect of this extract in animals with castor-oil-induced diarrhea occurred at doses of 200 and 300 mg/kg (72.7 and 77.1%, resp.), indicating that the activity of CBO was not dose dependent at the doses tested when castor oil or MgSO_4_ was used as the cathartic agent. The castor-oil-induced diarrhea model is suitable for the study of both secretory diarrhea and intestinal motility. Castor oil or its active component, ricinoleic acid, increases peristaltic activity and causes changes in the permeability of the intestinal mucosal membrane to electrolytes and water [16]. Additionally, the diarrhea induced by castor oil has been found to release prostaglandins [17] and to act on tachykinin receptors (NK1 and NK2) [16] and nitric oxide [18–20].

The osmotic properties of MgSO_4_ prevent the reabsorption of water and ions, leading to increases in the volume of the intestinal content. This compound also promotes the production of cholecystokinin from the duodenal mucosa [21], further increasing the secretions and exerting a positive motor effect on the small intestine [8, 21]. The activity of CBO in mice with diarrhea induced with AA or PGE_2_ is shown in Table 2. The effect of a 200 mg/kg dose of CBO on diarrhea induced by AA was 62.5%, an effect that diminished at 100 mg/kg (45.8%). CBO at these two doses had no effect on diarrhea induced by PGE_2_. The results of this inhibition study suggest that CBO affects the release of prostaglandins [22]. 

CBO did not demonstrate any effect on the defecation of untreated mice. 

**Figure 1:**
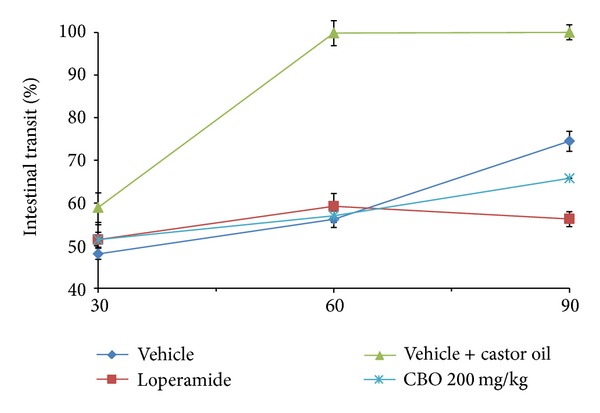
Effect of CBO (200 mg/kg) on intestinal transit in rats. The results are expressed as the mean of 5 determinations ± standard error. *P < 0.05 with respect to the castor-oil-treated group.

The intestinal transit of untreated rats, castor-oil-treated rats, and CBO-treated (200 mg/kg) rats is shown in Figure 1. The percentage of intestinal transit observed in CBO-treated rats with respect to castor-oil-treated rats after 30, 60, and 90 min was 51.4%, 57%, and 68.4%. These results indicate that CBO inhibited intestinal motility. 

**Figure 2:**
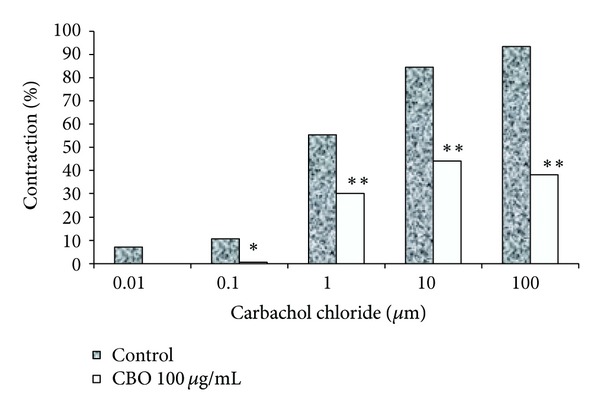
Effect of CBO (100 *μ*g/mL) on ileum contractions stimulated with carbachol chloride. The results are expressed as the mean of 5 determinations ± standard error. *P < 0.05 and **P < 0.01 with respect to the carbachol-chloride-treated group.

Carbachol chloride, at micromolar concentrations, causes a concentration-dependent contraction of the ileum isolated from rats. CBO (100 *μ*g/mL) had a significant inhibitory effect on the ileum stimulated with carbachol chloride at concentrations of 0.1 to 100 *μ*g/mL (Figure 2), possibly due to the interaction of this extract with the carbachol chloride muscarinic receptor (*μ*3) [23, 24]. The results of the present investigation demonstrate that CBO has antidiarrheal and antimotility activity in the experimental models studied.

CBO did not demonstrate toxic effects at the doses tested.

For this reason, CBO was separated by column chromatography. Eighteen fractions were obtained and compared by thin layer chromatography, which reduced the extract to 6 fractions. The antidiarrheal effect of these six fractions was tested on mice with castor-oil-induced diarrhea and the major effect was observed with F4 (90.1%), which was separated by chromatography, obtaining 14 fractions, which were compared by thin layer chromatography to obtain 7 fractions. The active fraction was FR5, which inhibited diarrhea induced by castor oil by 92.1%. These results are similar to those of F4, and the yield of FR5 was very low, so we proceeded with the study with F4.

The antidiarrheal effect of F4 on mice with diarrhea induced by AA or prostaglandin E_2_ was also tested. We found that F4 at doses of 100 mg/kg did not have an effect on diarrhea induced by prostaglandin E_2_; however, in diarrhea induced by AA, the inhibition was 72.9%. These results suggest that F4 inhibits the biotransformation of arachidonic acid to prostaglandin, as suggested by Manning et al. and Rodríguez-Lagunas et al., as AA is a substratum of prostaglandins [25, 26]. 

The composition of F4 and FR5 was obtained by CG-MS, and the results are shown in Table 3. We found that F4 has 76 components, the main compounds of which are palmitic acid (9.14%), linoleic acid (13.12%), trans-oleic acid (9.24%), stearic acid (3.39%), lignoceric acid (3.55%), octacosanoic acid (3.14%), and behenic acid (3.23%). FR5 has 7 compounds, four of which are palmitic acid, linoleic acid, trans-oleic acid and stearic acid; for this reason, we tested only these four fatty acids. 

The results of the antidiarrheal activities of these four components individually, a mixture of all four components, and F4 are shown in Table 4. At a dose of 25 mg/kg, linoleic acid inhibited diarrhea by 53.2%; this effect is the same as what is found at doses of 50 and 100 mg/kg. Similar results were observed at doses of 6.25 to 100 mg/kg when palmitic acid was tested; the maximum effect was at a dose of 100 mg/kg (67.5% inhibition). Stearic acid showed activity only at a dose of 100 mg/kg (43.9%). Oleic acid at doses of 1.56 and 3.12 mg/kg had no effect. The effect of oleic acid was 54.5% at a dose of 6.25 mg/kg, which did not change when the dose was further increased. For the mixture of the four acids (at the relative proportions detected in F4) at a dose of 1.56 mg/kg, the inhibition was 29.3%, reaching 70% at a dose of 100 mg/kg, which is higher than that obtained with any other fatty acid tested. These results suggest that there could be a synergic effect of the components of F4.

Linoleic acid has been previously reported to have activity on coronary diseases, hypertension, cancer, and rheumatoid arthritis [27]. Palmitic acid helps to maintain low blood levels of serum cholesterol and low-density lipoproteins and can help avoid some immunological responses [28]. There are no scientific reports regarding the antidiarrheal activity of these two acids, and we also did not find any reports about the pharmacological activities of stearic or oleic acid with respect to *B. odorata*. 

## 5. Conclusions

The present study validates the use of *B. odorata* in folk medicine as a remedy for intestinal cramps and diarrhea and suggests that this plant could possibly be used for the production of a phytomedicine.

Further pharmacological studies will be necessary to propose the exact mechanism of action.

## Figures and Tables

**Table 1:** Antidiarrheal effect of the aqueous, chloroform, and methanol extracts of *B. odorata *on mice with castor-oil-induced diarrhea^a^.

Treatment	Doses mg/kg	Percentage of inhibition ± s.e.
Vehicle	0.1 mL	—
Aqueous extract	200	43.8 ± 3.6*
Methanol extract	200	35.6 ± 5.8*
CBO	200	72.7 ± 5.3*
Loperamide	2.5	81.5 ± 4.5

^a^The results are the mean of 15 animals ± standard error.

*Significant difference was found: *P* < 0.05.

**Table 2:** Antidiarrheal activity of CBO on mice with castor-oil-, MgSO_4_-, arachidonic-acid-, and prostaglandin-E_2_-induced diarrhea^a^.

Cathartic agent	Treatment	Doses mg/kg	Percentage of inhibition
Magnesium sulphate	Vehicle	1 mL	0.0
	CBO	50	66.7 ± 6.7*
		100	84.9 ± 3*
		200	69.7 ± 6.0*
	Loperamide	2.5	87.5 ± 3.3

Castor oil	Vehicle	1 mL	0.0
	CBO	50	17.54 ± 4.6
		100	45.6 ± 6.3*
		200	72.7 ± 5.3*
		300	77.1 ± 1*
	Loperamide	2.5	89.77 ± 5.4

Arachidonic acid	Vehicle	0.1 mL	0.0
	CBO	100	45.8 ± 4.8*
		200	62.5 ± 4.5*
	Loperamide	2.5	75.3 ± 0.3

Prostaglandins E_2_	Vehicle	0.1 mL	0.0
	CBO	100	0.0
		200	0.0
	Loperamide	2.5	75.3 ± 0.3

^a^The results are the mean of 15 animals ± standard error.

^b^Significant difference was found: *P < 0.05.

**Table 3:** CG/MS results for the composition of F4 and FR5**.

Compound number	Retention time	Compound	Percentage composition F4	Percentage composition FR5
2	5.424	Lauric acid	0.08 ± 0.03	
5	6.427	n-Eicosane	0.03 ± 0.00	
11	8.055	Myristic acid	0.05 ± 0.01	
15	9.041	Pentadecanoic acid	0.26 ± 0.04	
20	9.829	Palmitoleic acid	0.25 ± 0.04	
21	10.206	Palmitic acid	15.03 ± 1.08	51.6540.977
24	11.046	Margaric acid	1.27 ± 0.07	
26	11.783	Linoleic acid	13.12 ± 0.39	32.379
27	11.852	Oleic acid	9.24 ± 1.73	
28	12.04	Stearic acid	3.39 ± 0.11	2.76
29	12.846	Nonadecanoic acid	0.09 ± 0.00	
30	13.386	(11E,13E)-11,13-Icosadienoic acid	0.20 ± 0.01	
31	13.446	(11E)-11-Icosenoic acid	0.25 ± 0.00	
32	13.72	Eicosanoic acid	1.89 ± 0.09	
33	14.5	Heneicosanoic acid	0.48 ± 0.01	
34	15.323	Behenic acid	3.23 ± 0.26	
36	16.042	Tricosanoic acid	1.44 ± 0.08	
37	16.531	n-Heptacosane	0.05 ± 0.01	
38	16.797	Lignoceric acid	3.55 ± 0.22	
40	17.388	Squalene	1.01 ± 0.08	
41	17.456	Pentacosanoic acid	0.63 ± 0.12	
43	18.159	Hexacosanoic acid	1.92 ± 0.11	
45	18.776	Heptacosanoic acid	0.30 ± 0.02	
46	19.205	Hentriacontane	0.20 ± 0.00	
47	19.47	Octacosanoic acid	3.14 ± 0.27	
54	20.687	Melissic acid	2.94 ± 0.19	
59	21.416	Friedelan-3-one	0.83 ± 0.39	
60	21.827	Dotriacontanoic acid	2.76 ± 0.23	
64	22.324	Tritriacontanoic acid	0.07 ± 0.01	
67	22.873	Tetratriacontanoic acid	1.13 ± 0.11	
76	27.466	Octacosyl palmitate	0.11 ± 0.01	

**Only identified compounds are presented. In F4, forty-five compounds were not identified; in RF5 seven compounds were detected and only four were identified.

**Table 4:** Effect of F4, palmitic, oleic, linoleic, and stearic acids, and their mixture at doses of 1.56 to 100 mg/kg on castor-oil-induced diarrhea.

Doses mg/kg	Diarrhea inhibition (%)					
	F4	Linoleic acid	Palmitic acid	Stearic acid	Oleic acid	Acids mixture
1.56	10.6 ± 4.7	31.7 ± 5.7	22.2 ± 6.9	26.3 ± 12.1	27.3 ± 1.8	29.3 ± 3.3
3.12	22.4 ± 3	38.1 ± 4.8*	30.2 ± 6.3	19.3 ± 4.6	20 ± 7.9	31.2 ± 1.5
6.25	20 ± 4.2	44.8 ± 4.6*	60.3 ± 7.5*	17.5 ± 9.3	54.5 ± 7.9*	27.5 ± 2.1
12.5	50.5 ± 6.1*	44.9 ± 1.7*	58.6 ± 6*	19.3 ± 4.7	52.7 ± 6.3*	53.2 ± 8.3*
25	67.6 ± 3.6**	53.2 ± 6.3*	60.8 ± 2.5*	12.3 ± 4.7	50.9 ± 6.5*	46.7 ± 4.2*
50	74.7 ± 1.0**	46.8 ± 7.6*	56.9 ± 1.3*	14.0 ± 3.6	52.7 ± 6*	47.7 ± 3.6*
100	90.1 ± 3.2**	42.1 ± 5.6*	67.5 ± 4.9*	43.9 ± 0.8*	58.2 ± 7.2*	70.6 ± 8.7**

The results are expressed as the mean of 15 animals ± standard error. *P < 0.05 and **P < 0.01 with respect to the C-group.
